# The application of next-generation sequence-based DNA barcoding for bloodmeal detection in host-seeking wild-caught *Ixodes scapularis* nymphs

**DOI:** 10.1186/s13104-021-05481-3

**Published:** 2021-02-18

**Authors:** G. A. Lumsden, E. V. Zakharov, S. Dolynskyj, J. S. Weese, L. R. Lindsay, C. M. Jardine

**Affiliations:** 1 Ontario Veterinary College, University of Guelph, 50 Stone Road E., Guelph, ON N1G2W1 Canada; 2 Canadian Centre for DNA Barcoding, Biodiversity Institute of Ontario, University of Guelph, 579 Gordon St., Guelph, ON N1G2W1 Canada; 3 Public Health Agency of Canada, National Microbiology Laboratory, 1015 Arlington St., Winnipeg, MB R3E 3R2 Canada; 4 Canadian Wildlife Health Cooperative, Ontario Veterinary College, University of Guelph, 50 Stone Road E., Guelph, ON N1G2W1 Canada

**Keywords:** Bloodmeal analysis, DNA barcoding, Blacklegged tick, Host identification, Next-generation sequencing

## Abstract

**Objective:**

Our objective was to apply next-generation sequence-based DNA barcoding to identify the remnant larval bloodmeals in wild-caught host-seeking (unengorged) *Ixodes scapularis* nymphs (*n* = 216). To infer host species identification, vertebrate DNA was amplified using universal primers for cytochrome *c* oxidase subunit I (COI) and sequenced using next-generation sequencing (NGS) for comparison against known barcode references.

**Results:**

Bloodmeal identification was unsuccessful in most samples (99% of 216 specimens) demonstrating a very low detection rate of this assay. Sequences that surpassed quality thresholds were obtained for 41.7% of nymphs (*n* = 90) and of those, confident species identification was obtained for 15.6% of nymphs (*n* = 14). Wild host identifications were only obtained from 2 specimens, where DNA from the eastern grey squirrel (*Sciurus carolinensis*) was identified. Human and bovine DNA was identified in remaining nymphs and considered to be contaminants. Further optimization of the technique is required to improve detection of remnant bloodmeals in host-seeking nymphs.

## Introduction

Bloodmeal analysis (BMA), an approach to investigate pathogen transmission, can identify the origin of bloodmeals by detecting the remnant host DNA within off-host ticks. Bloodmeal analysis in ticks has primarily used PCR-based techniques. A variety of genetic targets and post-PCR techniques have been explored with varying degrees of success in terms of host detection [1–6]. One BMA technique is DNA barcoding, a method of species identification based on short, standardized gene regions such as the cytochrome *c* oxidase subunit I (COI) mitochondrial gene (i.e., the barcode for animal species) [7]. COI is a well conserved gene across taxa with sufficient variation between closely related species, allowing for high resolution of species identification compared to other gene targets (e.g., cytochrome *b*, 12S rDNA, 18S rDNA) [7, 8]. By using universal vertebrate primers, DNA barcoding proved successful in engorged larvae and its extensive vertebrate barcode libraries identified all wild host species investigated [9]. However, DNA-based BMA remains unsuccessful in host-seeking, unengorged ticks where the last consumed bloodmeal could have been obtained weeks to over a year prior in the previous tick life stage resulting in significant degradation of target DNA [10, 11].

Next-generation sequencing (NGS) allows for high-throughput parallelization of sequencing reactions (deep sequencing) from low DNA concentrations and therefore may, when used in tandem with DNA barcoding [12], provide greater sensitivity to identify host DNA of low quantity and quality from host-seeking ticks. In a previous study, we found that the remnant bloodmeal in unengorged *I. scapularis* nymphs could be detected up to no later than two months after moulting using this technique (next-generation sequence-based DNA barcoding) [13]. The short temporal window of detection may have been impeded by an increased rate of bloodmeal digestion from laboratory rearing conditions [14] and application in wild tick populations may prove more successful. In the present study, we aimed to apply next-generation sequence-based DNA barcoding to identify the remnant larval bloodmeals in wild-caught host-seeking *I. scapularis* nymphs.

## Main text

### Methods

*Ixodes scapularis* were collected from Thwartway (44° 29′ 57.5″ N, 76° 15′ 02.6″ W) and Endymion Islands (44° 18′ 10.8″ N, 76° 05′ 53.4″ W), in Thousand Islands National Park Ontario, Canada in June 2017. Ticks were collected via tick dragging as described by Clow and colleagues [15]. Collected ticks were placed into 5 mL vials and kept alive for 1–2 days at room temperature thereafter they were frozen at − 80 °C. The life stage and species of each tick was determined using taxonomic keys [16].

Prior to DNA extraction, each frozen tick was washed using a two-step washing regimen of 70% v/v ethanol, followed by DNA-free water. Using the zirconium oxide beads, lysis buffer, and Tissue Lyser (Qiagen), the whole body of each tick was homogenized followed by overnight incubation, as outlined by (10.1093/jme/tjaa192) [13].

DNA of individual ticks was extracted at the Canadian Centre for DNA Barcoding (CCDB, Guelph, ON, Canada) using a silica-membrane based protocol (outlined in [13, 17]). Two negative controls consisting of only lysis buffer and one known host positive control tissue (*Oryctolagus cuniculus*) were processed with each plate in parallel with samples. All samples were randomly distributed across 96-well plates and all negative and positive controls were randomly distributed within a single plate.

The two-stage PCR amplification for NGS was conducted identically to the universal barcode assay methods (based on [18]; outlined in [13]), with the exception of controls. Briefly, the first round of PCR used a cocktail of degenerate primers tailed with M13 sequences designed to amplify the last 190 bp region of COI for 96 host species belonging to the classes Aves, Mammalia, and Reptilia native to Eastern Canada. Primer sequences are found in Table 1. The second round of PCR incorporated “fusion primers” (i.e., adapter sequences and unique molecular identifier tags) for characterization and sample-read identification by the Ion S5 sequencing platform (Thermo Fisher Scientific, Waltham, MA, USA) [19]. An additional negative control was included for each round of PCR consisting of PCR mix (Thermo Fischer Scientific) and 2 μL of Hyclone ultra-pure water (Thermo Fisher Scientific) and randomized within each 96-well plate (*n* = 6). Thermocycling conditions for the first and second round of PCR were identical to conditions used by Moran and colleagues [18].

**Table 1:** Cytochrome *c* oxidase subunit I primer sequences used for the species-specific and universal barcode assays each forward and reverse primer was tailed with an M13 forward and reverse sequence respectively

Cocktail name	Ratio	Sequence (5′–3′)	References
C_BloodmealF2_t1	1	TCATTACAACWATTATYAAYATRAA	Lumsden et al. (in press)
	1	TCATCACAACAGCAATYAAYATRAA	
Mod.Mam.Rev_t1	1	TTCTCAACCAACCACAAAGACATTGG	Ivanova et al. [17]
	1	TTCTCAACCAACCACAARGAYATYGG	
	3	TTCTCAACCAACCAIAAIGAIATIGG	

All PCR 2 products, including PCR replicates, were sequenced on the Ion S5 platform, with each replicate sequenced individually. Samples were prepared for sequencing by adding 1010 μL of water to 5 μL of 1 ng/μL purified product for a concentration of 26 pM. Library preparation and chip loading was performed on the Ion Chef System (Thermo Fisher Scientific). Samples were sequenced using a 530 v.1 chip according to the manufacturer’s instructions. The resultant data were processed through an identical bioinformatic pipeline [19] as outlined in [13]. After the filtering of low quality reads (QV < 20), primer and adapter sequences were removed. Operational taxonomic units (OTUs) were formed from reads with 98% identity followed by the removal of low abundance artefactual and chimeric reads by any OTUs composed of less than 10 reads [20, 21]. A basic local alignment search (BLAST) against the “All BOLD Bin” barcode library was conducted (BOLD; http://www.boldsystems.org/index.php). Sequence taxonomic assignments were only considered genuine after meeting the following identification confidence thresholds: at least 95% sequence similarity (identity), a minimum overlap of query sequence with a reference sequence of ≥ 100 nucleotides, and a depth of coverage of at least 50 reads (i.e., total read count) [19]. All samples that yielded a sequence that met these identification confidence thresholds from one or both PCR replicates were considered as a host identification. Spurious assignments (i.e., non-North American species) were removed from the data set and deemed contaminants.

### Findings

**Fig. 1:**
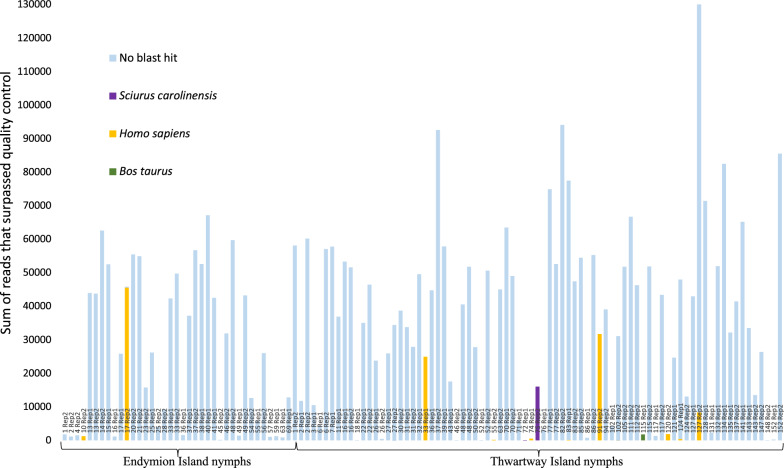
The sum of reads produced from both PCR replicates are depicted for wild-caught nymphs from Endymion and Thwartway Islands. With the exception of “no BLAST hit” sequences, the sum of reads depicted exceed the minimum identification confidence thresholds of 95% identity, a minimum overlap of query sequence to ≥ 100 nucleotides, and a depth of coverage of ≥ 50 reads for identification. All “no BLAST hit” sequences were unidentifiable by the “All BOLD Bin” reference library, likely attributed to non-target amplification. Amplified products from 51.57% (33/64) and 62.41% (98/157) of nymphs from Endymion and Thwartway respectively did not produce any reads that met the minimum confidence thresholds from either PCR replicate

**Fig. 2:**
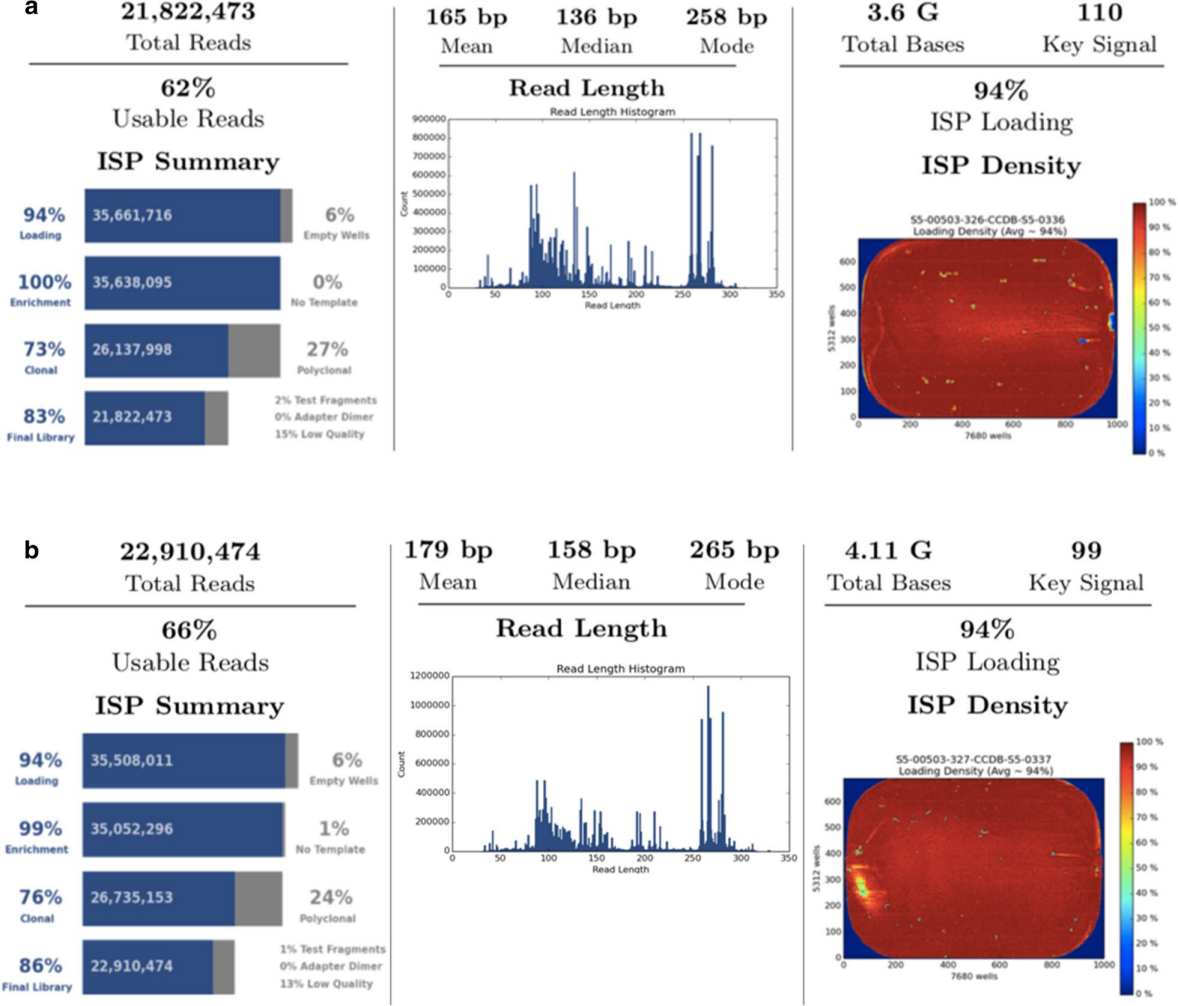
The Ion S5 sequencing performance metrics for all specimens with PCR replication 1 depicted in A and PCR replication 2 depicted in B. For PCR replicate 1 and 2, 94% of wells in a 530 v.1 chip were loaded with template Ion Sphere Particle (ISP) beads to generate a read. After automated processing of the bioinformatics pipeline, more than 21 million and 22 million sequencing reads were produced with an average read length of 165 bp and 179 bp, respectively

A total of 216 *I. scapularis* nymphs were collected; 152 from Thwartway Island and 64 from Endymion Island. Regardless of whether visible PCR product was observed following gel electrophoresis, all specimens (*n* = 216) and all controls (*n* = 15) from both PCR replicates were sequenced. A summary of host identifications that met the confidence thresholds for all 216 *I. scapularis* wild-caught nymphs is depicted in Fig. 1. Sequences that surpassed the quality threshold were obtained for 41.7% of nymphs (*n* = 90) and of those, a species identification that surpassed the confidence thresholds was obtained for 15.6% of nymphs (*n* = 14). One host species was identified as eastern grey squirrel (*Sciurus carolinensis*) from two Thwartway nymphs, 0.93% (*n* = 2/216) of specimens. Two other species identifications, *Homo sapiens* and *Bos taurus*, met the minimum confidence thresholds for 4.63% (*n* = 10/216) and 0.93% (*n* = 2/216) of specimens, respectively. The sequencing performance metrics for both replicates are shown in Fig. 2. Poor overall sequencing of target template is depicted in the read length histogram with high variation in sequence product length (i.e., non-specific products).

No DNA was detected in the negative controls that met the identification confidence thresholds except for *Homo sapiens* detected in one extraction control. All positive controls met the identification confidence thresholds in both PCR replicates and yielded the correct known species. Summary of results from all controls is shown in Additional file 1.

### Discussion

The next-generation sequence-based DNA barcode assay applied in the present study was unsuccessful in identifying the last bloodmeal consumed by almost all host-seeking *I. scapularis* nymphs and may reflect the inherent challenges testing unengorged ticks. Bloodmeal identification was unsuccessful in most samples (99% of 216 specimens) demonstrating a very low detection sensitivity (i.e., the proportion of ticks in which host DNA was positively identified) of this assay for host-seeking *I. scapularis* nymphs. In previous studies using various PCR-based techniques, a much higher detection sensitivity of bloodmeal identification (~ 50% [2–4, 22, 23]) was achieved in unengorged nymphs. However, it is difficult to compare host detection sensitivities between studies due to differences in sample type (e.g., tick species, tick life stage), time of sampling with respect to the likely time of last bloodmeal consumption and methodology (e.g., DNA extraction, PCR and post-PCR methods).

The degradation of the bloodmeal over time can have a large impact on the detection sensitivity of any tick BMA technique [24]. Decline in detectable host DNA over time and seasonal variation in detection has been demonstrated in multiple studies [1, 3, 4, 6]. In the present study, most nymphs were collected in June and likely consumed their last bloodmeal as larva the previous fall [25]. This timeline allows for an extensive duration of bloodmeal degradation and increased rates of bloodmeal digestion due to longer exposure of high temperatures and extended light to dark ratios than emerging spring or fall nymphs [14]. In previous studies, host detection was greater for host-seeking nymphs collected in the fall and spring compared to nymphs collected during summer [3, 4]. Targeted sampling of host-seeking ticks during time frames in which ticks have likely more recently consumed their last bloodmeal may improve recovery of host DNA.

Despite the overall low host detection in the present study, the remnant bloodmeal from two nymphs were identified as *S. carolinensis*. Detection of *S. carolinensis* DNA is plausible as this species inhabits the region and is commonly parasitized by *I. scapularis* [26]. It is possible that detection in those two nymphs was successful as they may have recently moulted from the larval stage allowing for less degradation of the host DNA within the remnant blood meal.

Contamination is an issue when using a degenerate assay on ticks with low quantity/quality DNA from unknown hosts [1, 27]. The detection of *H. sapiens* and *B. taurus* in numerous nymphs are likely a result of contamination. Even though both species are parasitized by *I. scapularis*, they could be confidently deemed as contaminants due to the geographic locations of where ticks were collected. *Bos taurus* are not present on either island locations and public access was prohibited on Endymion and limited on Thwartway during 2016 and 2017. Ultimately, contamination would pose a larger issue for BMA in wild ticks from areas that are more accessible.

A large proportion of unengorged nymph samples demonstrated an increased amplification of non-target DNA in which no species identification was assigned (i.e., no BLAST hit) (Fig. 2). The “no BLAST hit” results may be due to the amplification of non-target DNA competing with target DNA. A subset of “no BLAST hit” OTUs with the highest read counts were compared against an alternative reference database, the National Center for Biotechnology Information (NCBI), but an identification was still not obtained. Moving forward, increasing the annealing temperature and/or salt concentrations of the PCR could increase the specificity of the primers [28, 29]; however, the abundance of such non-target amplification within the dataset typically occurs when there is no other template to amplify [28]. Ultimately, increasing the annealing temperature and/or salt concentrations for PCR may remove false positive amplification but may not increase the sensitivity of host detection as starting host DNA template may simply remain insufficient.

Although the usefulness of DNA barcoding for BMA has been demonstrated in engorged larva [9], the lack of success in the present study may simply indicate that there is insufficient host DNA for detection due to DNA degradation of the bloodmeal over time. Moving forward, some potential options can be explored to improve the detection of the assay such as, (a) targeted tick sampling to early spring or in fall to reduce the amount of degradation to the last consumed bloodmeal, (b) re-optimize the conditions of PCR with universal primers (e.g., increase annealing temperatures and salt concentrations) to increase amplification specificity, and (c) reduce the degeneracy of primers by either using non-degenerate group-specific primers or targeting a different molecular marker that is more conserved than COI (i.e., cytochrome *b*). Employing these strategies will narrow the range of detectable species but ultimately, the trade-off of increasing the detection of remnant tick bloodmeals will still allow for valuable insights into vector-host assemblages.

## Limitations

- The technique herein described is not currently sufficient for BMA in host-seeking ticks and requires further optimization.
- BMA seems to be most effective in host-seeking ticks that have fresh blood remnants in their guts and degradation of host DNA likely increases in nymphs that have moulted months before their collection.
- Results generated by technique can be limited by vertebrate contamination when used on ticks from unknown hosts.

## Supplementary Information

**Additional file 1.** Summary of sequence results for all controls. Sum of reads for all controls (*n* = 15) ran in parallel with wild-caught nymphs for both PCR replicates. All negative controls failed in PCR replicate 2.

**Additional file 2.** Consolidated sequence results for all wild-caught nymphs. Consolidated sequence identifications generated after the bioinformatic pipeline for each wild-caught nymph including both PCR replicates.

## Data Availability

The consolidated sequence summary spreadsheet for each species identification for wild-caught nymphs are available as complementary material (Additional file 2).

## Abbreviations

BLAST: Basic local alignment search tool
BMA: Bloodmeal analysis
BOLD: Barcode of life database
COI: Cytochrome *c* oxidase subunit I
DNA: Deoxyribonucleic acid
NCBI: National Center for Biotechnology Information
NGS: Next-generation sequencing
PCR: Polymerase chain reaction
OUT: Operational taxonomic units
