# Supplementary material for: The application of next-generation sequence-based DNA barcoding for bloodmeal detection in host-seeking wild-caught Ixodes scapularis nymphs
Source: BMC Res Notes. 2021 Feb 18;14:67. doi: 10.1186/s13104-021-05481-3 (PMC7891142; doi:10.1186/s13104-021-05481-3)
Supplement: Supplementary file 1 — Additional file 1. Summary of sequence results for all controls. Sum of reads for all controls (n = 15) ran in parallel with wild-caught nymphs for both PCR replicates. All negative controls failed in PCR replicate 2. [file 13104_2021_5481_MOESM1_ESM.docx]

Table S1. Sum of reads for all controls (*n*=15) ran in parallel with wild-caught nymphs for both PCR replicates. All negative controls failed in PCR replicate 2.

| PCR replicate | Type of control | Sum of reads | Species identification |  |
| --- | --- | --- | --- | --- |
| 1 | Extraction negative | - | Failed |  |
| 1 | Extraction negative | - | Failed |  |
| 1 | Extraction negative | 10 | *Oryctolagus cuniculus* |  |
| 1 | Extraction negative | 209 | *Homo sapiens* |  |
| 1 | Extraction negative | - | Failed |  |
| 1 | Extraction negative | - | Failed |  |
| 1 | PCR 1 negative | - | Failed |  |
| 1 | PCR 1 negative | - | Failed |  |
| 1 | PCR 1 negative | 14 | *Oryctolagus cuniculus* |  |
| 1 | PCR 2 negative | - | Failed |  |
| 1 | PCR 2 negative | - | Failed |  |
| 1 | PCR 2 negative | - | Failed |  |
| 1 | Positive | 92082 | *Oryctolagus cuniculus* |  |
| 1 | Positive | 130947 | *Oryctolagus cuniculus* |  |
| 1 | Positive | 99100 | *Oryctolagus cuniculus* |  |
| 2 | Positive | 104013 | *Oryctolagus cuniculus* |  |
| 2 | Positive | 163898 | *Oryctolagus cuniculus* |  |
| 2 | Positive | 149682 | *Oryctolagus cuniculus* |  |
| *Samples with reads that met the minimum sequence thresholds | | | | |
